# Experimental Study on Damage Detection in Timber Specimens Based on an Electromechanical Impedance Technique and RMSD-Based Mahalanobis Distance

**DOI:** 10.3390/s16101765

**Published:** 2016-10-22

**Authors:** Dansheng Wang, Qinghua Wang, Hao Wang, Hongping Zhu

**Affiliations:** School of Civil Engineering & Mechanics, Huazhong University of Science and Technology, Wuhan 430074, China; M201473117@hust.edu.cn (Q.W.); wangh31@vanke.com (H.W.); hpzhu@hust.edu.cn (H.Z.)

**Keywords:** damage detection, timber specimen, electromechanical impedance, Mahalanobis distance, RMSD, experimental study

## Abstract

In the electromechanical impedance (EMI) method, the PZT patch performs the functions of both sensor and exciter. Due to the high frequency actuation and non-model based characteristics, the EMI method can be utilized to detect incipient structural damage. In recent years EMI techniques have been widely applied to monitor the health status of concrete and steel materials, however, studies on application to timber are limited. This paper will explore the feasibility of using the EMI technique for damage detection in timber specimens. In addition, the conventional damage index, namely root mean square deviation (RMSD) is employed to evaluate the level of damage. On that basis, a new damage index, Mahalanobis distance based on RMSD, is proposed to evaluate the damage severity of timber specimens. Experimental studies are implemented to detect notch and hole damage in the timber specimens. Experimental results verify the availability and robustness of the proposed damage index and its superiority over the RMSD indexes.

## 1. Introduction

In recent several decades, many nondestructive detection (NDD) techniques have been extensively applied to metal and concrete materials. However, studies on NDD of timber is rarely reported in the literature, while timber is one of the most popular construction materials in many countries. Since local damages such as hole, decay, and crack caused by the adverse environmental influence and external loads extensively exist in wooden structures, reliable damage detection techniques for timber or wooden structures are heavily needed. 

At present some conventional damage detection techniques have been applied on timber and wooden structures, for instance, modal parameter methods [1,2,3], the stress wave method [4], ultrasonic methods [5,6], and the soft capacitor method [7]. Philipp and Thomas [8] recently reviewed existing feasible methods to assess the integrity of glue-laminated timber elements. While a few of abovementioned methods can, somehow, quantitatively assess any damage, the majority of them can only provide qualitative information.

EMI is a powerful and promising damage detection technique for timber or wooden structures based on piezoelectric smart materials, which possess some advantages such as low cost and easy-to-implement. The most outstanding feature of the technique is its sensitivity to incipient structural damage. The EMI technique was initially presented by Liang et al. [9] to model the dynamic interaction between a host structure and a PZT transducer. Subsequently, the technique was applied to detect the damage of metal components and structures in the aerospace and mechanical engineering, and a great deal of experimental investigations have been successfully developed. For instance, the EMI method was employed to detect the structural integrity of assembled space trusses [10], local-area state monitoring of aircraft [11], health monitoring of aging aerospace structures [12], damage detection of aluminum specimens [13,14], and fault detection of rotating machines [15]. In recent years, the EMI method has drawn increasing attention in the area of civil engineering. For instance, Park et al. [16] performed damage detection studies on typical components of civil structures, such as concrete walls reinforced by composite, 1/4-scale bridge components and a pipe joint. Tseng et al. [17] performed experimental and numerical investigations on the impedance-based technique for damage detection of a plain concrete beam. Soh et al. [18] studied the applications of the EMI technique, including strength prediction and damage evaluation. Hu et al. [19] implemented an experimental study on damage detection of a two-storey concrete frame based on the EMI method. They studied the correlation between the evaluation index and the distance of PZT sensors away from the damage and derived their sensing region in concrete. Annamdas et al. [20] presented an embedded PZT transducer method in EMI-based health monitoring of concrete structures. Shin and Oh [21] implemented an experiment to monitor the strength development of concrete at the early age utilizing the surface bonded PZT. Wang and Zhu [22,23] utilized embedded PZT transducers to monitor the concrete strength gain based on the EMI technique. They carried out experimental and numerical studies on damage identification of a plain concrete beam [24]. Hu et al. [25] studied an EMI-based damage detection method for a concrete plate, in which four distributed PZT transducers were utilized. Karayannis et al. [26] presented an experimental study to assess the damage of the reinforcing bars in concrete by utilizing bonded piezoelectric patches and implementing an integration analytical method based on the EMI technique. The abovementioned studies of damage detection methods based on the EMI technique were mainly aimed at steel and concrete materials. However, compared to steel and concrete timber is an anisotropic biomaterial. The properties of different parts of timber, such as elastic modulus, moisture content, and damping, are commonly not uniform in the spatial and temporal dimension due to the changing growth conditions. In the EMI technique, the wave velocities of timber in different directions can be different and the reflected waves can be more complex than those of steel and concrete materials. Therefore, it is necessary to verify the availability of EMI technique for timber samples. Recently, Annamdas et al. [27,28] performed experimental studies to monitor the degradation of wood in the presence of acids and the stiffening effect owing to insertion of steel nails on wood blocks based on the EMI technique, respectively. To provide more detailed insights and conclusions, it is necessary to carry out more studies on timber or wooden structures.

In recent years, damage identification algorithms have been widely used in structural health monitoring. Zhou et al. [29] proposed a damage detection method based on Mahalanobis square distance (MSD) and carried out an experimental study on the damage detection of a steel beam to validate the effectiveness of the proposed method. Mustapha et al. [30] presented novel methods for detecting damage—outlier analysis (OA) based on Mahalanobis square distance, and a multi-layer perceptron (MLP) neural network—and performed experimental investigations on a thin rectangular plate to verify the validity of the method. Li et al. [31] developed a new detection approach for local variation based on transmissibility and developed numerical studies on transmissibility for damage detection in beam structures. In order to avoid the disadvantage of strict input requirements for MSD-based damage detection, Nguyen et al. [32] proposed a novel method of enhanced data generation scheme and demonstrated the effectiveness of this method through applications on the data from a benchmark structure. Dervilis et al. [33] used the experimental data obtained from the Z24 and Tamar Bridges to explain the approach to apply the robust regression on the data analysis of structural health monitoring. However, the conventional identification algorithms may have some limits on the frequency band, the identification results and so on; for example, the conventional Mahalanobis distance may be unstable due to the highly ill-conditioned inverse matrix. Therefore, there is a need to propose a novel algorithm to handle this problem. 

In this paper, experimental studies on two different sorts of timbers, i.e., Scots pine (*Pinus silvestris*) and Bangkirai *(Shorea laevis*), have been carried out to explore the damage sensitivity of EM impedance signal on timber. In addition, the conventional damage index, root mean square deviation (RMSD) is employed to evaluate the damage severity of the timber specimens. To evaluate the damage severities more accurately, a new damage index, Mahalanobis Distance (MD) based on RMSD, was proposed and used for the damage detection of timber specimens. 

## 2. Damage Detection Methodology Based on EMI 

### 2.1. EMI Method

When a PZT sensor is subjected to mechanical stress, the surfaces of the sensor will produce electric charges. Contrarily, a PZT sensor will experience mechanical strain when subjected to an electric voltage. These phenomena are called piezoelectric effects. For the excitation and sensing PZT patches, the constitutive relation can be described by the following equations [9]:

(1) $$S_{i}=s_{ij}^{E}T_{j}+d_{mi}E_{m}$$

(2) $$D_{m}=d_{mi}T_{i}+\varepsilon_{mk}^{T}E_{k}$$

where *S* represents the strain, *D* represents the electric displacement, *T*, *E*, *S*, *ε* and *d* represent the stress, the electric field, the mechanical compliance and the piezoelectric constant, respectively. The subscripts *i*, *j*, *m* and *k* denote the direction of stress, strain, electric field, respectively. The superscripts *T* and *E* represent the conditions where no stress is applied and constant electric fields are applied, respectively. Equations (1) and (2) describe the converse and direct piezoelectric effect, respectively.

The EMI technique is based on the converse and direct piezoelectric effects of PZT. A PZT patch performs roles of both sensor and actuator simultaneously in the EMI technique. In this technique, a local vibration wave is produced when a PZT patch bonded to the surface of the base structure is stimulated by a sinusoidal sweep signal. As a result, the base structure vibrates along with the PZT patch. Subsequently, the vibration echo wave of the base structure will interact with the PZT patch and induce its electrical response. The electrical response is collected and analyzed by using an impedance analyzer, and then the electrical admittance signals are obtained. With the development of the damage, the properties of the base structure will change. By acquiring the electrical admittance signals of PZT patch and comparing those to the baseline signals, the damage of the base structure can be monitored. Therefore, the EMI technique can detect whether the damage appears in the structure or not.

The electromechanical admittance *Y*(*ω*) (the converse of *Z*(*ω*)) of the PZT patch is an integrated function of the mechanical impedance of PZT impedance sensor *Z_a_*(*ω*) and the mechanical impedance of the base structure *Z_s_*(*ω*), which can be formulated as:

(3) $$Y(\omega )=j\omega a\left[{\bar{\varepsilon }}_{33}^{T}(1-j\delta )-\frac{Z_{s}(\omega )}{Z_{s}(\omega )+Z_{a}(\omega )}d_{31}^{2}{\bar{Y}}_{11}^{E}\right]$$

where *d*_31_ represents the piezoelectric constant, ${\bar{Y}}_{11}^{E}$ represents Young’s modulus, and ${\bar{\varepsilon }}_{33}^{T}$ represents the complex dielectric constant of PZT at zero stress, respectively; *δ* is the dielectric loss tangent of PZT, and *a* is a geometric constant. The equation indicates that the electromechanical admittance of a PZT bonded to a base structure is directly correlated to the mechanical impedance of the base structure.

### 2.2. RMSD Index

To evaluate the extent of structural damage, the conventional damage index, RMSD, is often used in the EMI technique, which is expressed as Equation (4):

(4) RMSD=∑i=1N[Re(Yi)−Re(Y0)]2/∑i=1N[Re(Y0)]2

where *Y* represents the electrical admittance of PZT transducers, *Re* represents the real part of the electrical admittance, *N* represents the number of the measurement, subscript 0 and *i* represent initial and operational states, respectively.

For undamaged structures, the RMSD value is very close to 0, if there is no existence of other uncertainty but for the damaged structures the admittance curves obtained are different from the curves obtained from original undamaged structures. The value of RMSD increases as the damage severity increases.

Although the damage index, RMSD, is widely used, it still possesses some disadvantages as it can only characterize the correlation between two variables in a single frequency range at a time and cannot evaluate structural damage considering multiple frequency ranges at the same time. When the EM admittance in a certain frequency range is insensitive to structural damage, misjudgment will be induced by using the RMSD index.

### 2.3. Mahalanobis Distance Index Based on RMSD

To evaluate the level of damage more accurately, a new index, Mahalanobis distance (MD) based on RMSD, is introduced. MD describes the distance of covariance between two variables, as formulated in Equation (5), which provides an effective approach to calculate the similarity between two variables:

(5) $$MD={\left(x-\mu \right)}^{T}S^{-1}(x-\mu )$$

where *x* represents the multi-dimensional sample set, *S* represents the covariance matrix of the sample set, and *μ* represents the mean vector.

By analyzing the calculation procedure of MD based on RMSD, it can be found that the inverse matrix of *S* is highly ill-conditioned resulting in instability of the MD results. Therefore, an improved computation method for MD based on the RMSD of real EM admittance data is proposed to handle the ill-conditioned problem:

(6) 1≤r≤(p−1)

(7) $$\lambda_{1}\geq \lambda_{2}\geq \cdots \geq \lambda_{r}>\lambda_{r+1}=\cdots =\lambda_{p}=0$$

(8) $$MD^{2}(y,X)={\left(y-\bar{\mu }\right)}^{T}(P_{1}+TP_{2})(y-\bar{\mu })=\sum_{k=1}^{r}\frac{{\left(y_{k}-\mu_{k}\right)}^{2}}{\lambda_{k}}+\sum_{k=r+1}^{p}t_{k}{\left(y_{k}-\mu_{k}\right)}^{2}$$

where $P_{1}=E\left[\begin{array}{cccc} \lambda_{1}^{-1} & 0 & \cdots & 0\\ 0 & \lambda_{2}^{-1} & \cdots & 0\\ \cdots & \cdots & \cdots & \cdots \\ 0 & 0 & \cdots & \lambda_{r}^{-1} \end{array}\right]E^{T}$, $P_{2}=I_{p}-EE^{T}$, and $T=\left[\begin{array}{cc} I_{r} & 0\\ 0 & A \end{array}\right]$ are an adjustment matrix, $I_{r}$ is an r order unit matrix. $A=\left[\begin{array}{cccc} t_{r+1} & 0 & \cdots & 0\\ 0 & t_{r+1} & \cdots & 0\\ \cdots & \cdots & \cdots & \cdots \\ 0 & 0 & \cdots & t_{p} \end{array}\right]$, $t_{k}(k=r+1,\cdots ,p)$ is an adjustment coefficient, and it is often equal to $\frac{1}{\lambda_{r}}$.

$\mu ∊R^{p\times 1}$ and $S∊R^{P\times P}$ are assumed to be the mean vector and covariance matrix of the sample *X*, respectively. The rank of *S* meets Equation (6). *λ_i_* and *e_i_* are the i order eigenvalue and eigenvector of *S*, respectively, and the eigenvalues meet Equation (7). In addition, *E* = (*e*_1_, *e*_2_, *e*_3_, …, *e_r_*) is the base vector, which consists of the eigenvectors corresponding to the positive eigenvalues. Then the square of MD between the point $y∊R^{p}$ and the sample *X* is as formulated in Equation (8).

## 3. Experimental Study

### 3.1. Experimental Setup 

**Figure 1:**
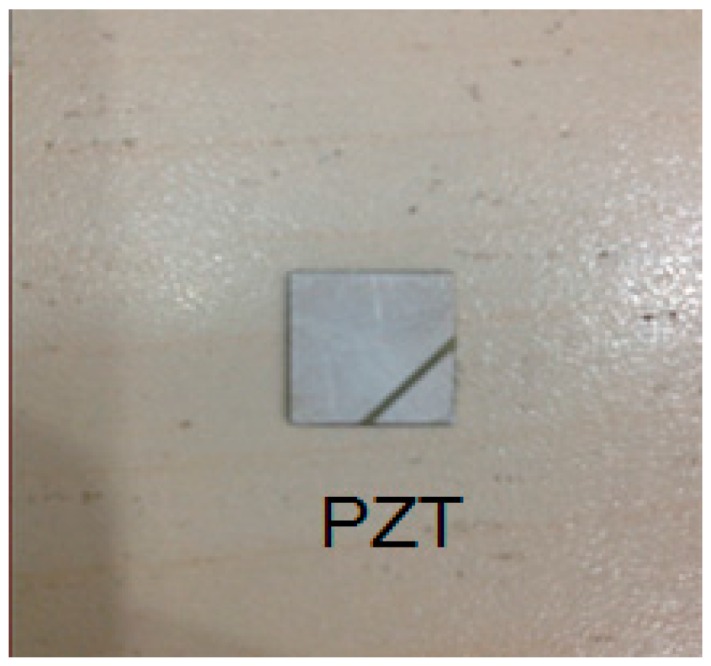
Configuration of the PZT patches.

Two different sorts of timbers, *Pinus sylvestris* and Bangkirai, representing conifer and hardwood, respectively, were selected for the test. Nine specimens for each kind of timber with different damage types were tested and monitored by using PZT patches with reversal electrodes, as shown in Figure 1. Each specimen had a length of 0.2 m, a width of 0.09 m, and a thickness of 0.02 m, and the dimensions of the PZT patches were 10 mm × 10 mm × 0.5 mm.

**Figure 2:**
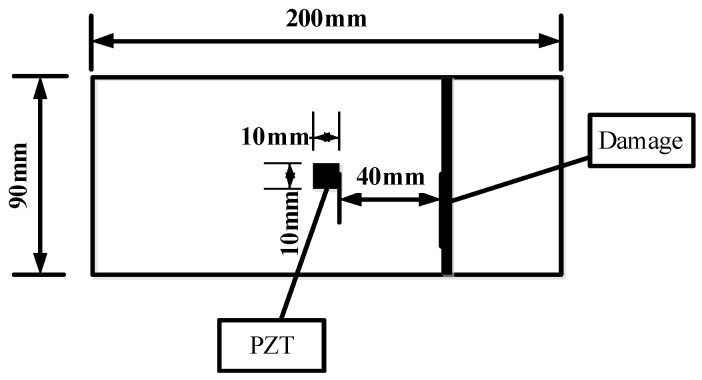
Sketch of timber specimens in group A.

**Figure 3:**
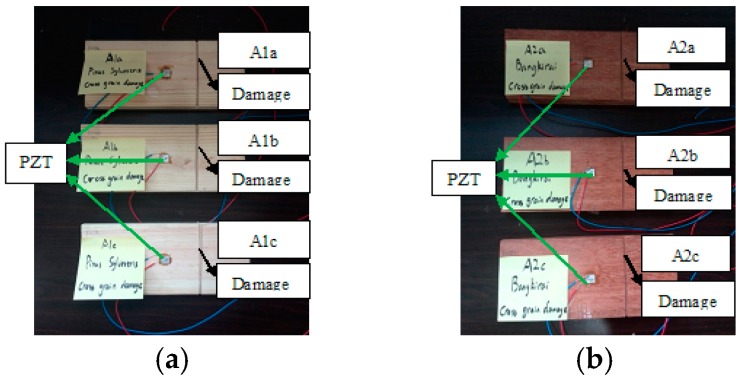
Timber specimens in group A (**a**) Pinus sylvestris and (**b**) Bangkirai.

**Figure 4:**
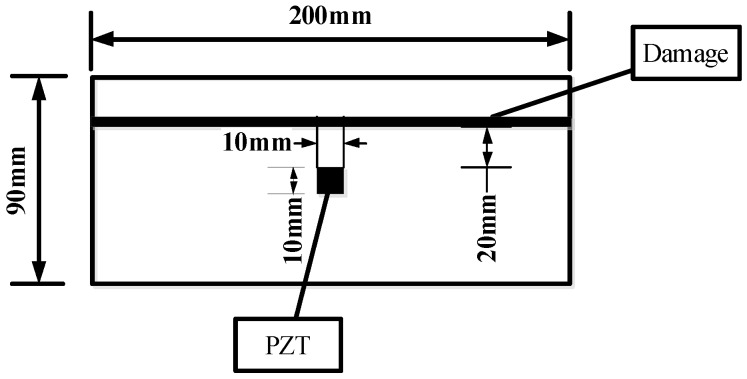
Sketch of timber specimens in group B.

**Figure 5:**
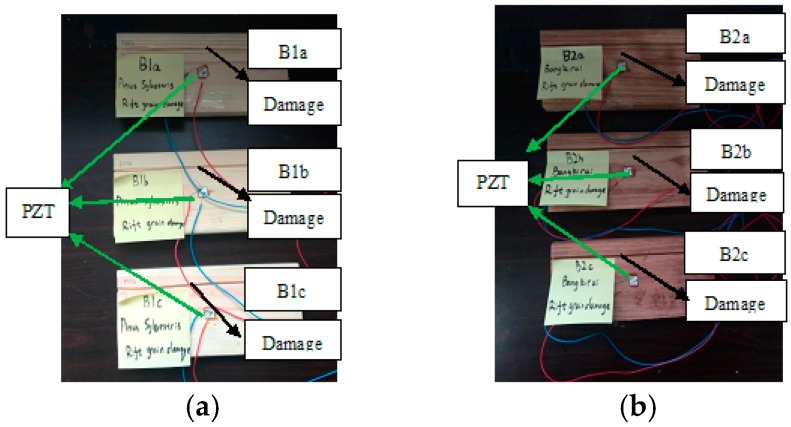
Timber specimens in group B (**a**) Pinus sylvestris and (**b**) Bangkirai.

**Figure 6:**
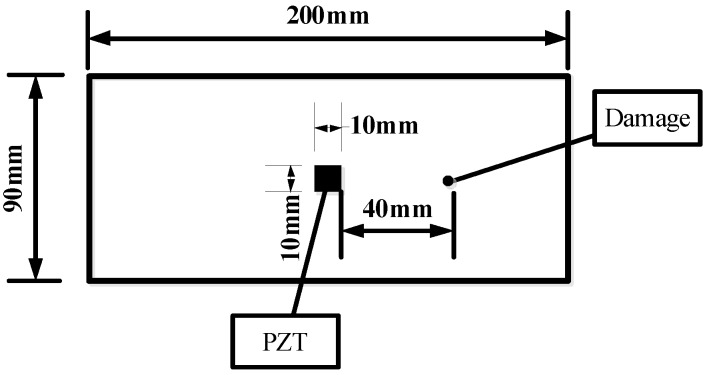
Sketch of timber specimens in group C.

**Figure 7:**
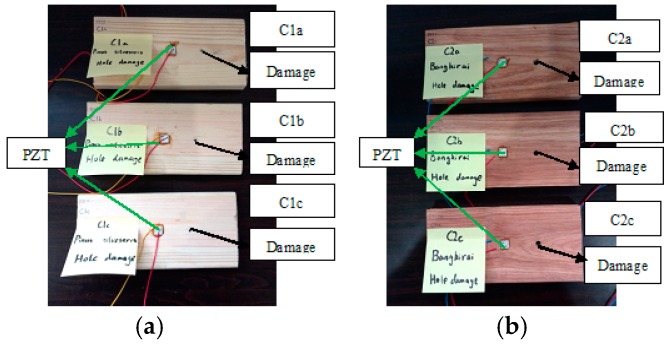
Timber specimens in group C (**a**) Pinus Sylvestris and (**b**) Bangkirai.

Three different damage types, namely, notch across the grain, notch along the grain and hole for group A, group B and group C, were involved in this study. A total of six identical specimens with three from the conifer and three from the hardwood were considered in each group. One PZT transducer was located at the middle of each timber specimen. In group A, a notch across the grain was fabricated at a distance of 40 mm away from PZT transducer, as shown in Figure 2. Three *Pinus sylvestris* specimens were numbered as A1a, A1b, and A1c, and the other three Bangkirai specimens were numbered as A2a, A2b and A2c, as shown in Figure 3. For specimens in group B, notch damage along the grain at a distance of 20 mm away from PZT transducer was made, as shown in Figure 4. Three *Pinus sylvestris* specimens were numbered as B1a, B1b, and B1c, and the other three Bangkirai specimens were numbered as B2a, B2b and B2c, as shown in Figure 5. For specimens in group C, a circular hole at a distance of 40 mm from PZT transducer was made, as shown in Figure 6. Three *Pinus sylvestris* specimens were numbered as C1a, C1b, and C1c, and the other three Bangkirai specimens were numbered as C2a, C2b and C2c, as shown in Figure 7. For each group of experiment, four types of damage were made. For group A and group B, the four notch damage examples corresponding to damage depths of 0, 2, 4 and 6 mm was considered respectively, and the width of the notch was about 3 mm. For group C, four hole damage cases corresponding to damage diameters of 0, 3, 6 and 8 mm was considered, respectively. The details of the specimens of groups A, B, and C are tabulated in Table 1, Table 2 and Table 3, respectively.

**Figure 8:**
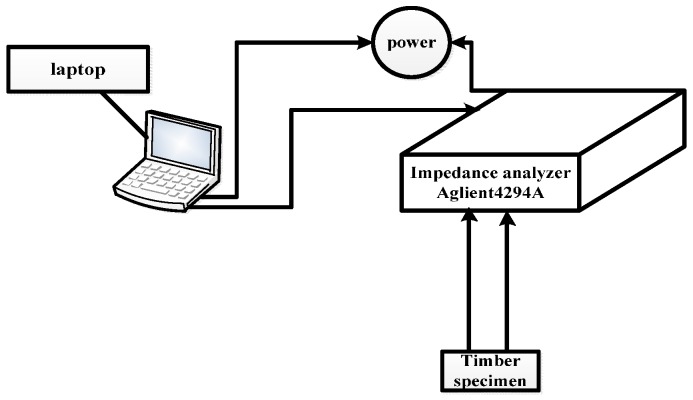
The flow chart of the experiments.

In the study, an Agilent 4294A impedance analyzer was used to measure the EM admittance curves of the PZT transducers. The sweep frequency range of the Agilent 4294A begins at 40 Hz and ends at 110 MHz. The PZT transducers were excited by 1 Vrms alternating voltage output. Four sweep frequency ranges, 40 Hz–30 kHz, 30–50 kHz, 50–150 kHz and 150–500 kHz, were selected as operation frequencies. A flow chart of the experimental setup is shown in Figure 8. The EM admittances of the PZT transducers were measured five times in each damage case to calculate the MD index values. In addition, to ensure the accuracy of the experiment, the experiment was carried out in a short time, in which the temperature and humidity were relatively stable (temperature was 25 °C, and humidity 68%), and there was no interference of external excitation sources. 

### 3.2. Experimental Results and Analysis

#### 3.2.1. Experimental Results 

The real admittances of PZT transducers bonded on different timber specimens were obtained in four different frequency ranges, respectively. In order to limit the length of the paper, only some parts of the results are shown here.

**Figure 9:**
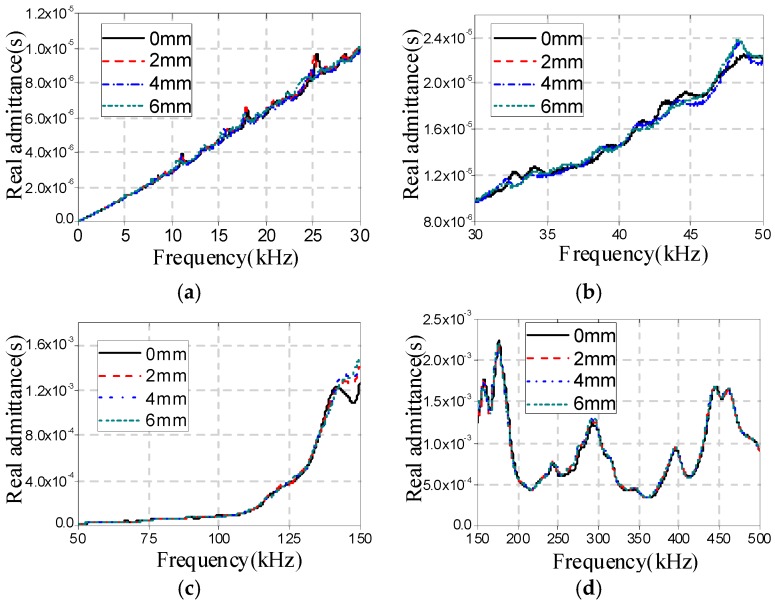
Real admittance for A1a in different cases (**a**) 40 Hz–30 kHz; (**b**) 30–50 kHz; (**c**) 50–150 kHz; and (**d**) 150–500 kHz.

**Figure 10:**
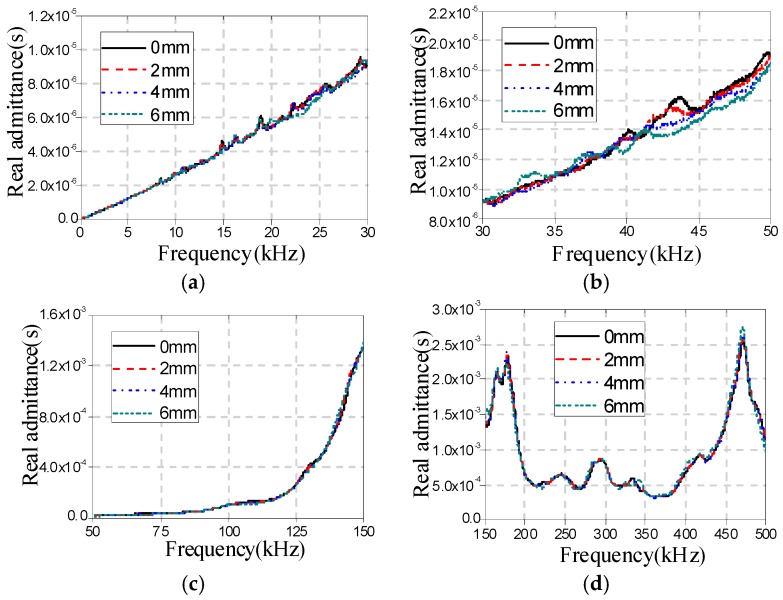
Real admittances for B1a in different cases (**a**) 40 Hz–30 kHz; (**b**) 30–50 kHz; (**c**) 50–150 kHz; and (**d**) 150–500 kHz.

**Figure 11:**
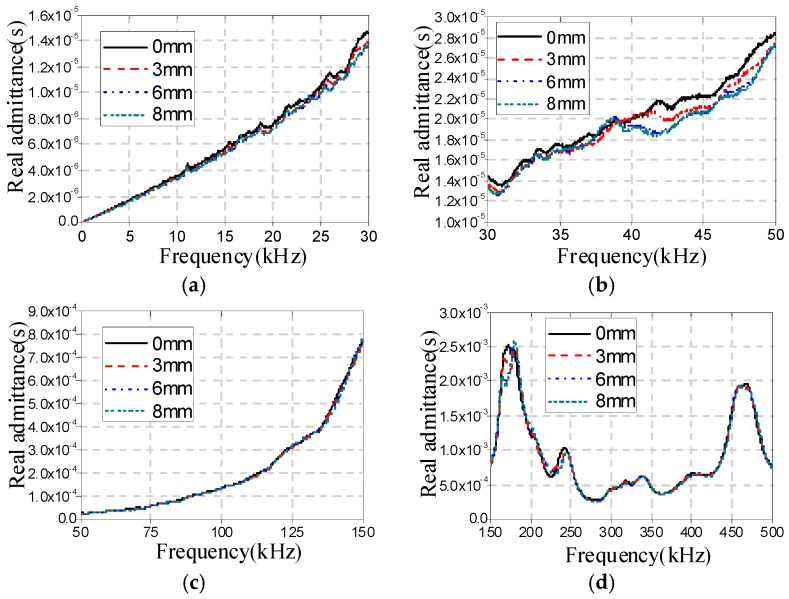
Real admittances for C1a in different cases (**a**) 40 Hz–30 kHz; (**b**) 30–50 kHz; (**c**) 50–150 kHz; and (**d**) 150–500 kHz.

Figure 9, Figure 10 and Figure 11 show the real admittance curves for specimens A1a, B1a, and C1a, respectively, in different cases. From these figures it can be found that the changes in the real admittance curves in the frequency range of 30–50 kHz are most obvious among all four frequency ranges with the increase of damage severity. In addition, it is also found that by only observing the changes in real admittance curves it is hard to distinguish the sensitivity of the EMI technique to the three different types of damage. 

#### 3.2.2. Experimental Analysis Based on RMSD Index Values

**Figure 12:**
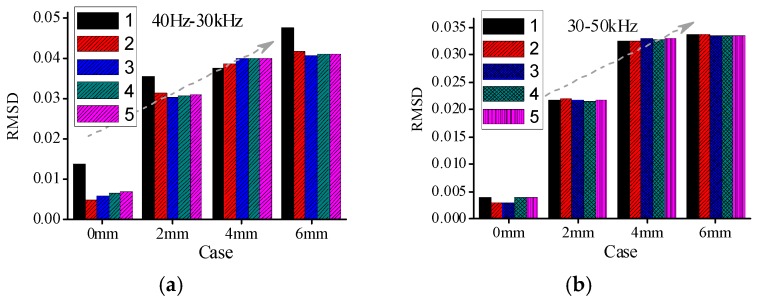

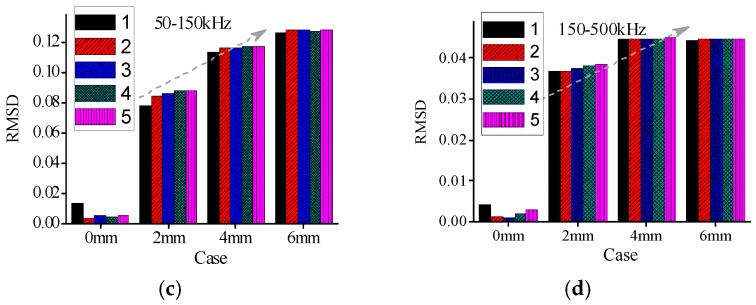
RMSD values for A1a in different cases (**a**) 40 Hz–30 kHz; (**b**) 30–50 kHz; (**c**) 50–150 kHz; and (**d**) 150–500 kHz.

**Figure 13:**
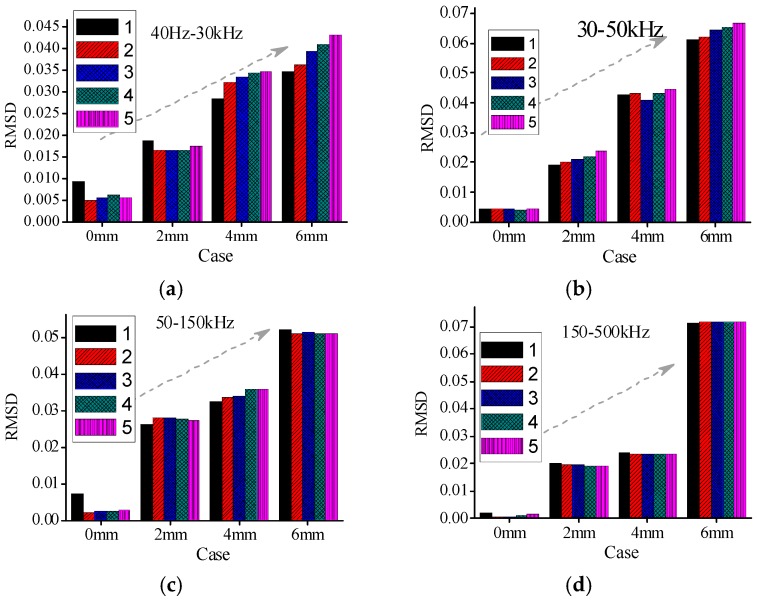
RMSD values for B1a in different cases (**a**) 40 Hz–30 kHz; (**b**) 30–50 kHz; (**c**) 50–150 kHz; and (**d**) 150–500 kHz.

**Figure 14:**
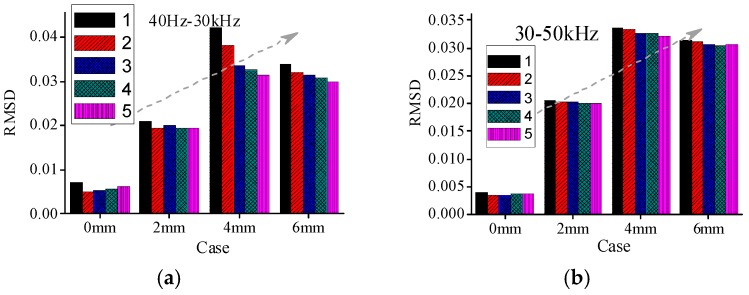

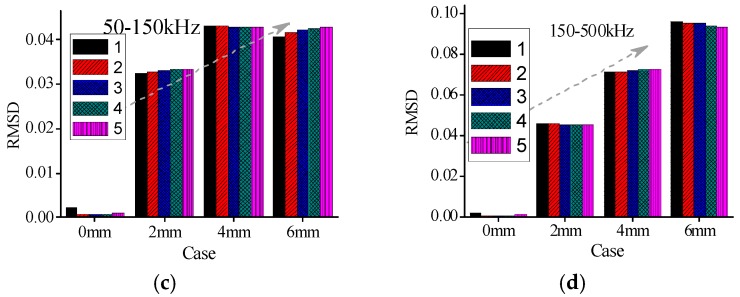
RMSD values for C1a in different cases (**a**) 40 Hz–30 kHz; (**b**) 30–50 kHz; (**c**) 50–150 kHz; and (**d**) 150–500 kHz.

To evaluate the severity of damage, the RMSD index, based on real experimental admittance data in four different frequency ranges are calculated, respectively. The mean value of five repeated measurements in case 1 (intact state) is taken as the reference value. The RMSD index values are obtained in different cases for all timber specimens respectively. In this paper, The RMSD index results for A1a, B1a, and C1a specimens are shown in Figure 12, Figure 13 and Figure 14, respectively. From the figures it can be found that the RMSD index values become larger with the increase of damage severity. 

However, there are some misjudgments in different frequency ranges for all the three groups of specimens when the RMSD index is used to evaluate the damage severities of timber specimens. In addition, the changes of the RMSD index are different in different frequency ranges. From the RMSD results it is difficult to determine which frequency range is relative better than the other frequency ranges for different timber specimens. 

Table 4 shows the capability of RMSD index to identify the damage severities in different frequency ranges for all timber specimens. From Table 4, it can be found that there are some misjudgments when RMSD index is used to evaluate the damage severity for different groups of specimens. For example, for specimen A1a in group A, the RMSD index values in the frequency ranges of 30–50 kHz and 150–500 kHz failed to indicate the true damage severities. 

Similarly, the RMSD index values for A2c also failed to reveal the true damage severities in the frequency ranges of 50–150 kHz and 150–500 kHz. Therefore, it is necessary to seek a more reliable damage index to evaluate the damage severity of timber samples.

#### 3.2.3. Experimental Analysis Based on MD Index

**Figure 15:**
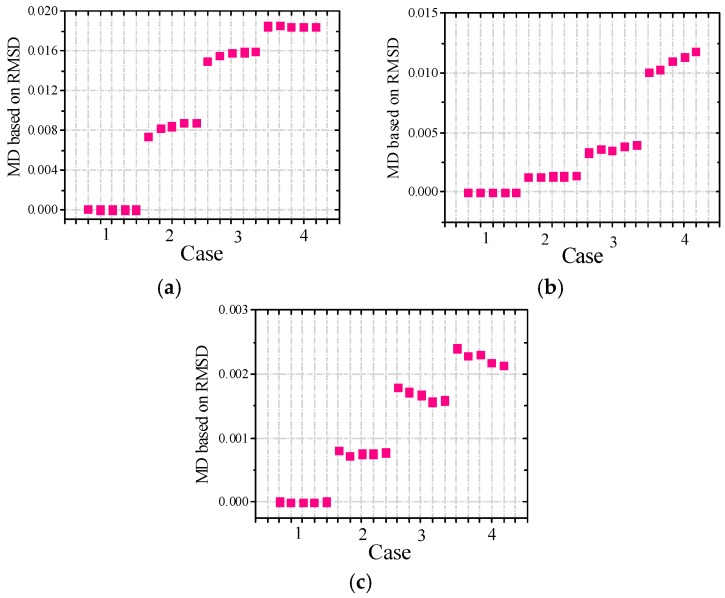
MD values based on RMSD for (**a**) A1a; (**b**) B1a and (**c**) C1a.

**Figure 16:**
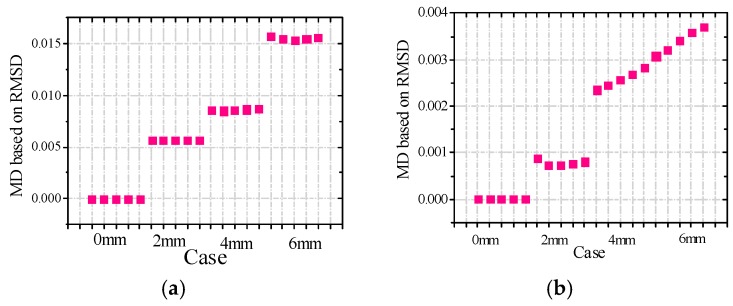

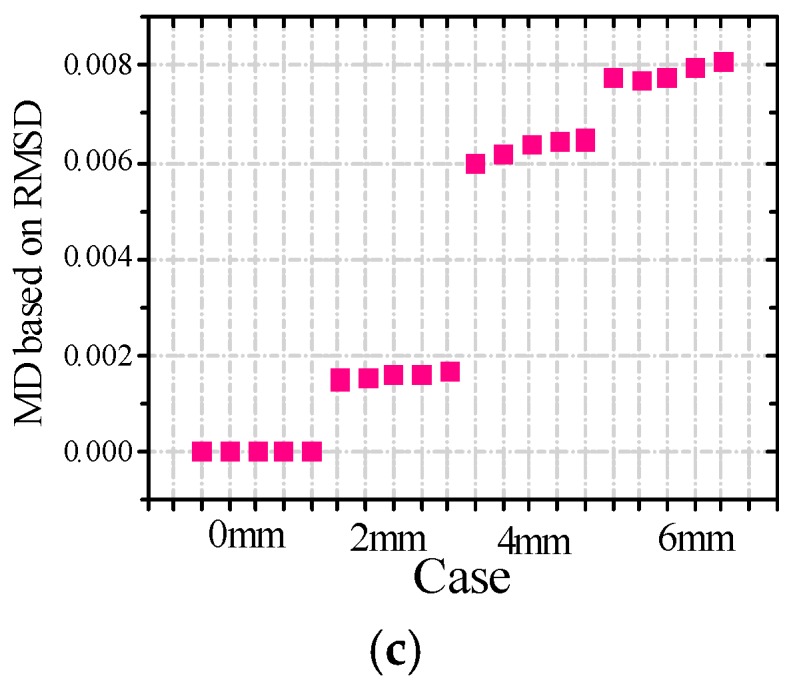
MD values based on RMSD for (**a**) A2a; (**b**) B2a and (**c**) C2a.

To reliably evaluate the damage severity of timber specimens, the MD index values based on RMSD are computed for all three groups of specimens. The MD values based on RMSD considering the above-mentioned four frequency ranges are shown in Figure 15 for specimens A1a, B1a and C1a, and in Figure 16 for specimens A2a, B2a and C2a. The MD indexes for all the remaining timber specimens successfully identify the damage severity.

From the obtained MD values for all timber specimens, it can be found that the MD values increase generally with the increase of damage severity. From Figure 15 and Figure 16, it is found that the EMI technique is more sensitive to the notch damage across the grain compared with the notch damage along the grain and hole damage. This is because the main propagation direction of waves is along the grain, so the main propagation is disturbed when the notch is across the grain and as a result, more waves with the damage information are reflected. Therefore the EMI technique is relatively more sensitive to notches across the grain compared to notches along the grain and holes. 

More importantly, there is no misjudgment for the two types of timber specimens in different cases when the RMSD-based MD index is used to evaluate the damage severities. This indicates the capability of the MD index based on RMSD is robust and reliable to evaluate the damage severities of timber specimens with different damage types. The RMSD-based MD index can avoid the disadvantages of the conventional RMSD index depending on a single frequency range.

## 4. Sensitivity of the EMI Technique Using the RMSD-Based MD

**Figure 17:**
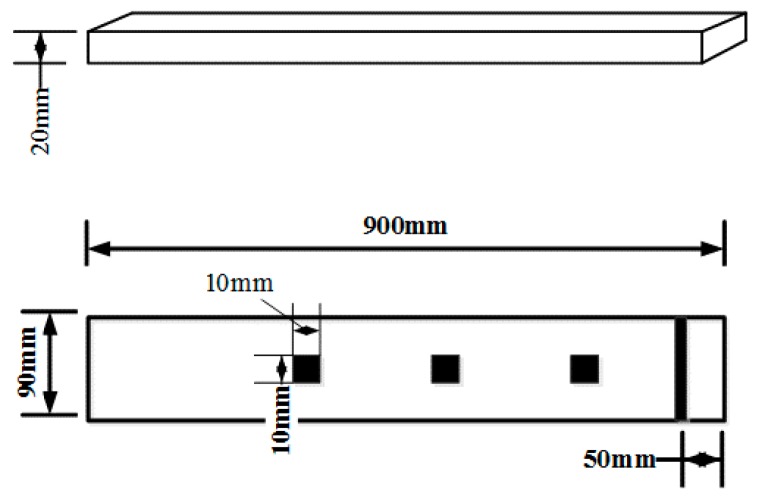
Sketch of timber specimen for the sensitivity verification experiment.

**Figure 18:**
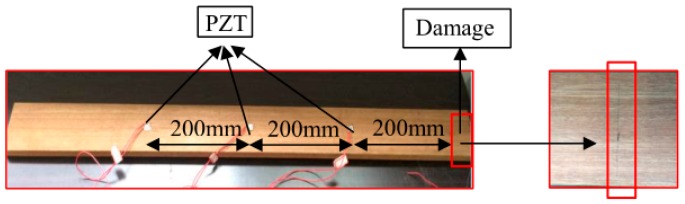
Timber specimen for the sensitivity verification experiment.

In order to explore the performance for varying flaw distances from the PZT and the sensitivity of the EMI technique to incipient structural damage, a specimen of Bangkirai representing hardwood was selected for the test. The specimen had a length of 900 mm, a width of 90 mm, and a thickness of 20 mm, and the dimensions of the PZT patches with reversed electrodes were 10 mm × 10 mm × 0.5 mm. A notch at a distance of 50 mm from the end of the specimen was made across the grain. In order to simulate incipient structural damage, the notch was cut slightly with a knife in the experiment. The damage depths were approximately 0.3, 0.6 and 0.9 mm, respectively and the damage width was approximately 0.3 mm. The details of the specimens are shown as Figure 17 and Figure 18. In the experiment, the PZT transducers were set at a distance of 200, 400 and 600 mm from the damage, and numbered as 1, 2, and 3, respectively. 

The EM admittance curves of PZT transducers were obtained through the Agilent 4294A impedance analyzer and the PZT transducers were excited by 1 Vrms alternating voltage output. Four sweep frequency ranges, 40 Hz–30 kHz, 30–50 kHz, 50–150 kHz, and 150–500 kHz, were selected as test frequencies. The EM admittances of PZT transducers were measured five times in each damage case. The real admittances of PZT transducers bonded on different locations were obtained. 

**Figure 19:**
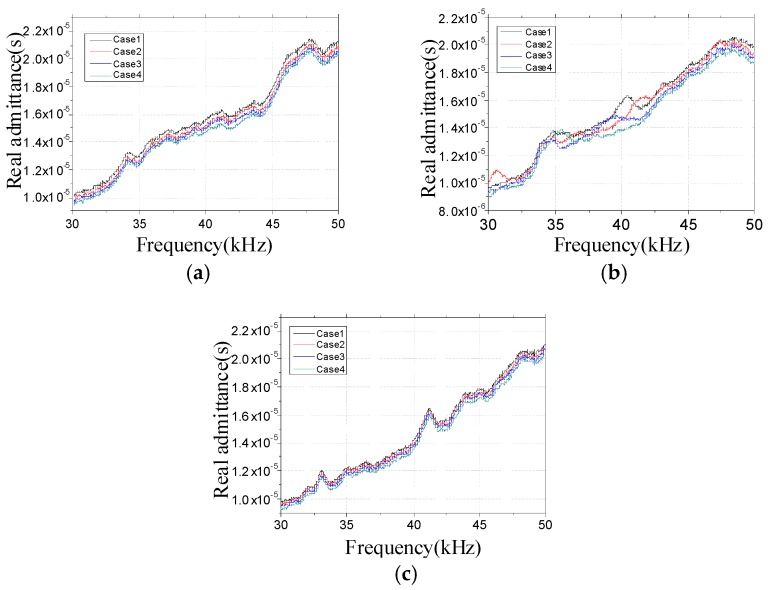
Real admittances for all cases in the frequency range of 30–50 kHz (**a**) PZT1; (**b**) PZT2; and (**c**) PZT3.

**Figure 20:**
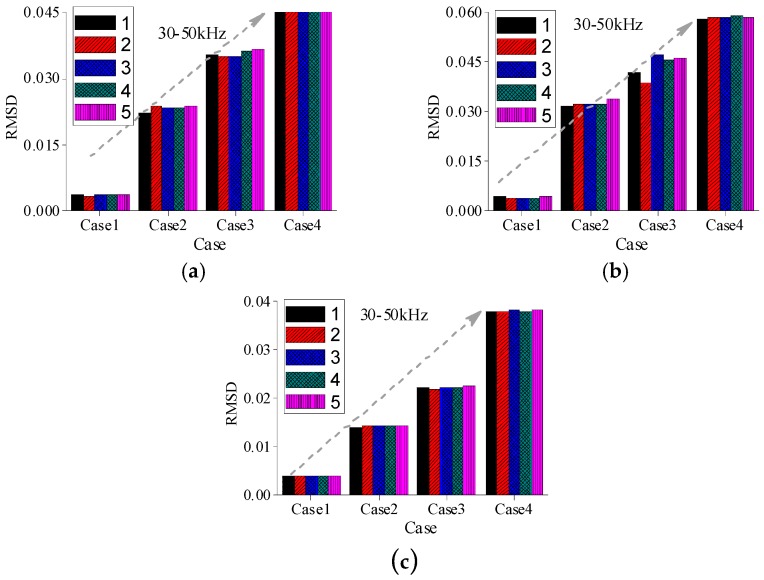
RMSD values for all cases in the frequency range of 30–50 kHz (**a**) PZT1; (**b**) PZT2; and (**c**) PZT3.

**Figure 21:**
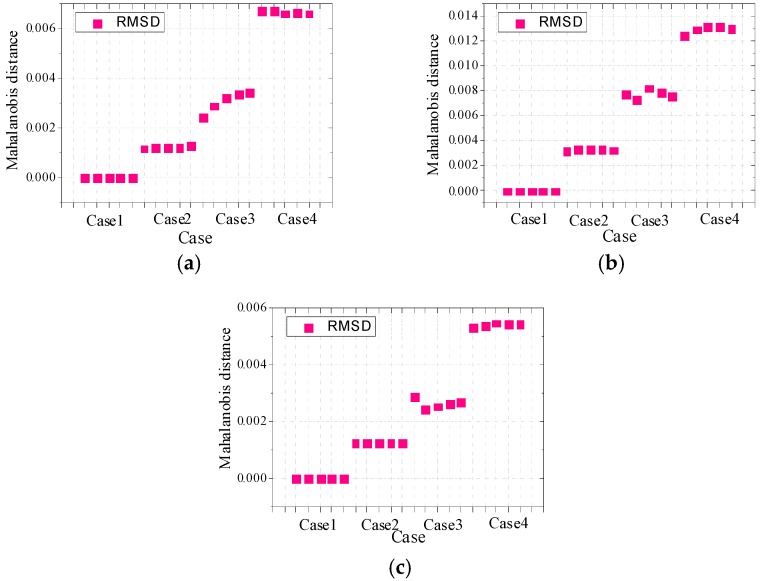
MD values based on RMSD for (**a**) PZT1; (**b**) PZT2 and (**c**) PZT3.

Since the real admittances in the frequency range of 30–50 kHz are most sensitive to damage among all above-mentioned frequency ranges, only the real admittance curves in the frequency range of 30–50 kHz for PZT 1, PZT 2, and PZT 3 are shown in Figure 19. From Figure 19, it is found that the changes of admittance curves in the frequency range of 30–50 kHz are obvious when the incipient damage is presented. Figure 19 also shows that PZT 1 and PZT 2 are more sensitive to the incipient damage and its expansion than PZT 3. In other words, with the increasing distance between the damage and the PZT, the sensitivity of the PZT impedance transducer to damage declines. To evaluate the severity of damage quantitatively, the RMSD indexes were calculated here, as shown in Figure 20. From Figure 20, it is found that the RMSD index values become larger with the increase of damage severity. In addition, The MD indexes based on RMSD for the timber specimens are also calculated, as shown in Figure 21.

From Figure 21, it is found that the MD index values decline with the increase of distance away from the damage, in other words, the changes of electrical admittance signals become insensitive with the increasing distance between the damage and the PZT. Therefore, from the point of view of implementation, the EMI technique could be used to detect incipient damage of timber specimens, but the PZT transducers need be installed in some sort of dense grid to ensure efficiency in the important positions.

## 5. Conclusions

In this paper, the EMI technique is used to detect the damage of timber specimens, and a new damage index, MD based on RMSD, is proposed to evaluate the damage severity of test specimens. Experimental studies on damage detection of two types of timber specimens are implemented to verify the validity of the EMI technique and the proposed index. From the experimental results, it can be concluded that the EMI technique is both effective and sensitive to the local damage of the timber specimens for damage detection, especially in the frequency range of 30–50 kHz. More importantly, it can be concluded that the EMI technique combined with RMSD-based MD index is effective and reliable to identify the severity of incipient damage, and the MD index seems be more sensitive to the notch damage across the grain than the other two types of damage. In addition, it is also concluded that the proposed RMSD-based MD index is more reliable than the conventional RMSD index method for damage quantification.

## Figures and Tables

**Table 1:** Experimental set in group A.

Number	Timber Types	Dimensions (mm)	Damage Type	Damage Cases
A1a A1b A1c	Pinus sylvestris	200 × 90 × 20	notch across the grain	0, 2 mm, 4 mm, 6 mm
A2a A2b A2c	Bangkirai	200 × 90 × 20	notch across the grain	0, 2 mm, 4 mm, 6 mm

**Table 2:** Experimental set in group B.

Number	Timber Types	Dimensions (mm)	Damage Type	Damage Cases
B1a B1b B1c	Pinus sylvestris	200 × 90 × 20	notch along the grain	0, 2 mm, 4 mm, 6 mm
B2a B2b B2c	Bangkirai	200 × 90 × 20	notch along the grain	0, 2 mm, 4 mm, 6 mm

**Table 3:** Experimental set in group C.

Number	Timber Types	Dimensions (mm)	Damage Type	Damage Cases
C1a C1b C1c	Pinus sylvestris	200 × 90 × 20	hole	0, 3 mm, 6 mm, 8 mm
C2a C2b C2c	Bangkirai	200 × 90 × 20	hole	0, 3 mm, 6 mm, 8 mm

**Table 4:** Capability of RMSD index.

Specimens		40 Hz–30 kHz	30–50 kHz	50–150 kHz	150–500 kHz
**Group A**	**A1a**	√	×	√	×
	**A1b**	√	√	√	√
	**A1c**	√	√	√	√
	**A2a**	√	√	√	√
	**A2b**	√	√	√	√
	**A2c**	√	√	×	×
**Group B**	**B1a**	√	√	√	√
	**B1b**	√	√	√	√
	**B1c**	√	×	×	√
	**B2a**	√	√	√	√
	**B2b**	×	×	√	√
	**B2c**	√	√	×	√
**Group C**	**C1a**	×	×	×	√
	**C1b**	√	√	√	√
	**C1c**	√	√	√	√
	**C2a**	√	√	×	×
	**C2b**	×	×	×	√
	**C2c**	√	×	×	√

Where “√” represents the recognizable case; “×” represents the case where it fails.
